# The Diseases of the Army of Occupation in the Summer of 1846

**Published:** 1847-03

**Authors:** H. R. Robards

**Affiliations:** Memphis, Tennessee


### WESTERN JOURNAL

OF

### MEDICINE AND SURGERY.

MARCH, 1847.

### Art. I.—*The Diseases of the Army of Occupation in the Summer of* 1846. By H. R. Robards, M.D., of Memphis, Tennessee.

The following abstract from notes taken while the writer was acting as Surgeon to a regiment of volunteers from Tennessee on its march to Mexico, and during a few weeks while stationed at Matamoras, is presented to the profession, in the hope that its details will be found interesting and useful. The notes were penned in camp, and my short military experience has taught me that the camp is not a place for easy or finished composition.

As early as May last, in anticipation of a call for volunteers for the war then just breaking out with Mexico, the stirring notes of the fife and drum were heard in Memphis; in a few weeks, five companies were made up, and remained in organization until the final lots were drawn in June, when only three were admitted into service.

The requisition made upon Tennessee was for three regiments—two of infantry, and one of cavalry; the latter, to which I was attached, was ordered to rendezvous at Memphis by the 15th of June, or as soon thereafter as possible. This regiment consisted of three companies from East, five from Middle, and two from West Tennessee. I am particular in locating the different companies, in order to show the varying influences of a southern climate upon constitutions from different sections of the State.

Three companies only had arrived at Camp Carroll, the place designated for the encampment, two miles east of Memphis, on the day appointed; on the 24th of June, the whole number were there; and on the 27th of July, we took up the line of march for Mexico; the entire distance, about fifteen hundred miles, to be traveled by land.

Our camp near Memphis, so far as could be observed, was was free from local causes of disease; the situation was elevated, and both men and horses were furnished with excellent water from a single large spring. The weather, during a greater portion of the time, was excessively warm and dry, and the roads and whole encampment disagreeably dusty. For the first three weeks after the arrival of the troops at this place, we had little else, in the way of disease, to contend with but diarrhœa, intermittents, colic, etc., brought on chiefly by exposure and imprudence in diet. The companies from West Tennessee, being acclimated, suffered least; two-thirds of the men from other parts of the State were attacked with one or the other form of these diseases soon after their arrival. The treatment was simple, and only varied with the cause. If diarrhœa, and the cause imprudence in diet, a dose of castor oil relieved most of them; if it did not, broken doses of calomel, ipecac. and morphia invariably put a stop to it. This was a favorite combination with me in this disease, and throughout the entire march never failed, when the patient could be restrained in his diet. The cases of intermittent fever were cured with equal certainty by administering *two doses of quinine, of ten grains each*, one given six hours before the chill, and the other two. As colic was often brought on by gorging with indigestible food, emetics were frequently administered, and gave instant relief. If the indications did not call for this remedy, calomel and opium, followed by a brisk purgative, were given; and if, as is often the case in this disease, an operation could not be procured in time by purgatives, then I had recourse to an expedient which with me has never failed, and I have used it in numerous cases of obstruction from various causes: this is forcing large quantities of lukewarm water into the intestines by injection. If the obstruction is caused by impacted fæces, the water reaches them, they are moistened, and pass off. If it be partial hernia, as I have known in one instance, where a knuckle of the intestine is retained in the internal ring, the gradual and powerful pressure of the water draws the bowel out, straightens it, and thereby removes the obstruction. I have often been astonished at the immense quantity of water that can be forced into the bowels without the slightest injury to the patient, but often with the effect of affording instant relief.

About the 20th of June, a most troublesome camp disease broke out. I refer to measles, which, notwithstanding every precaution that could be used, remained among the men three months; there were not less than three hundred and fifty cases of the disease, and yet all recovered. The treatment adopted was chiefly expectant. Sometimes the patients took stimulating diaphoretics, and often during the march drank freely of hot whisky toddy, even while the fever was on, without any bad effects that could be perceived; *purgatives were decidedly injurious in any form.* Diarrhœa, which often occurred as a sequel to this disease, was sometimes obstinate, but yielded to external irritants, mucilaginous drinks, anodynes, etc.

About the first of July, a few cases of continued fever occurred in camp ; but they were confined to one company, which was stationed in an elevated part of the encampment, and in which the usual degree of cleanliness was observed. This company was from a region in Middle Tennessee noted for its insalubrity; two of these cases proved fatal, in consequence, I believe, of the dread of the hospital that existed in that company, which deterred the men from calling for medical aid until the disease had made considerable progress. The brain was the organ that suffered most, delirium being a constant attendant. The treatment consisted of cold applications to the head, blistering, alteratives, etc. One fact I observed in the management of this fever, which strikes me as worth recording; that is, that *quinine invariably aggravated all the symptoms.* If experience should prove this to be always the case, will it not go far to prove that the cause of intermittents and remittents differs from that of continued fever?

During the time we were at Camp Carroll, the companies from East Tennessee suffered most from diarrhœa, colic, and such other diseases as are brought on by exposure and imprudence in diet, their constitutions seeming as yet to resist the causes of fever in any of its forms. It will be remembered that East Tennessee is for the most part a high, broken and healthy section of the State. In the companies from Middle Tennessee there were many cases of remittent fever, at the same time that diarrhœa, colic, etc. were common. The two companies from West Tennessee suffered but little from any other disease except intermittent fever.

From the close proximity of the camp to town, and the freedom with which soldiers, when unrestrained, are known to indulge in every kind of dissipation, the number of cases of syphilis and gonorrhœa which I have to report will not seem extravagant, namely: twenty-three of the former, and eighty-four of the latter.

The first stages of syphilis were treated with calomel in combination with sarsaparilla; without waiting for the gums to be touched, this form of mercury was laid aside after a few days, and the cure completed by giving in full doses the proto-iodide of mercury, and sometimes the hydriodate of potassa. The latter was always used when the gums had been previously touched with mercury; dry lint or calomel was applied to the chancres, and iodine ointment to the buboes. The patients generally recovered in a few days.

Gonorrhœa was treated with equal success, by administering two parts of the comp. extr. buchu and one of balsam copaiba in teaspoonful doses three times a day, in the first stage; in the second, that is, after using the above four or five days, injections of a strong solution of nitrate of silver or acetate of lead were employed, and generally effected a cure at once.

On the 20th of July, our encampment was changed to the bank of the Mississippi river opposite Memphis. After this, we had but one or two cases of continued fever, while the intermittents and remittents assumed a much more malignant form.

But before I proceed farther, it may be as well to state that a little upwards of a thousand men, either belonging to or in some way attached to the regiment, came under the surgeon's care; out of which number, on the 4th of October, there had been more than twelve hundred cases reported. Of course, some of the men were several times on the sick list; but the case was never reported unless it was a different disease from the one previously treated; relapses were never reported the second time. This regiment, as I have before mentioned, consisted of volunteers from different sections of the State, of various professions and callings; many were educated gentlemen; many belonged to the respectable class of farmers and mechanics, and not a few were loafers. With all, the habits, customs, diet, etc. of a camp life were different from what they had before known. The sickly season was just approaching when they arrived at Memphis; the country through which they traveled was one of the sickliest in the United States. Is it wonderful, then, that so large a number of cases should have occurred? Is it not astonishing that only five had died up to the 4th of October, when I temporarily left the regiment? It will be admitted that this success was almost if not entirely unprecedented, and I attribute it greatly to the efficacy of a single remedy, quinine. This was our sheet-anchor, and, without it, my opinion is that our regiment, now by far the largest and most efficient in the service, would have been completely disabled.

From the accumulated experience of the profession, it is clear that quinine acts in some manner specifically upon the nervous system, producing, as I have lately seen, when given at improper times and in over doses, complete derangement of that system. May this not throw some light upon the vexed question as to the organ primarily affected by the remote cause of fever? If quinine cures fever, and the action of the article is specifically upon the nervous system, does it not follow that the cause of fever must act primarily upon that system? But I return from this digression to my notes.

I mentioned before that the remittent and intermittent fevers assumed a more malignant form on the bank of the river, but did not state that diarrhœa almost entirely disappeared from the camp, except when induced by drastic purgatives, which was so common an occurrence that we had to guard scrupulously against their use.

The symptoms that ushered in intermittent or remittent fever were very similar, and such as usually occur in that section of country. However threatening the premonitory symptoms might be, it was not often the case that medical aid was called for until the patient found himself shivering with a chill; then he was let alone until the stage of excitement passed off. If at this time the tongue, skin, etc. indicated a very disordered state of the secretory organs, a mercurial in some form, in combination with a gentle purgative, was given during the sweating stage; the kind of purgative to be given was always suggested by the nature of the case, the constitution of the patient, etc. Very often, indeed, they were not used at all, and the cure proved equally effectual. We usually commenced with the quinine eight or ten hours before the chill ought to return; ten grains were given, and repeated four hours afterwards; the third dose was given or not as the case seemed to require.

Ten grains of quinine I regard as a maximum dose, *if to be repeated.* I however frequently gave as much as thirty grains; but it was always when the case was seen for the first time a few hours before the chill was expected, and it constantly kept it off. I prefer giving the remedy in ten grain doses, for the reason that its effects are always certain and it acts more effectually upon the skin, and not so frequently as an emetic as when given in larger doses. My manner of giving it was simply to mix it with water. To some this is a bitter, nauseous dose, and caused vomiting at once; then it had to be made into pills, but I think the solution much more effectual and rapid. The doses I have mentioned invariably put a stop to the chill, whether simple or malignant; but to guard more certainly against a return, we usually ordered five or ten grains, according to circumstances, every morning for three or four days. If, after the chill was checked, the secretions remained vitiated, an alterative was given at night, and repeated if necessary.

The cases of remittent fever were treated differently, and it is in the treatment of this disease that quinine is most frequently abused. It is very much the custom in the South, at this time, to administer quinine in all the stages of fever and in large doses. My experience justifies me in saying that such a course is not only unnecessary, but altogether unjustifiable. In my opinion, quinine should never be given unless the remissions are very distinct, and *never in the hot stage.* I usually, in this form of fever, commenced the treatment, after the first exacerbation had passed off, by administering a gentle purgative, in combination with some article that would keep up an action upon the skin without irritating the stomach and bowels. Ipecacuanha generally answered the purpose remarkably well. Most frequently the combination was of calomel and ipecac., given in broken doses, which course, varied as circumstances required, was kept up for two or three days, when the remissions usually became more distinct, and quinine had the effect of arresting the disease. These cases rarely continued on hand longer than four or five days.

There are some other diseases which many southern physicians regard as having a malarious origin, and treat indiscriminately with quinine; I allude more particularly to dysentery and diarrhœa. Now, I have not found such practice judicious, but, if I mistake not, have seen very injurious consequences result from it. But when these diseases assume, as they sometimes do, a periodical form, then quinine, in combination with morphia, is the remedy. I should never recommend it in these diseases unless they did assume such a form. This combination I have found exceedingly useful in the treatment of other diseases attended with the nervous irritability very common in the fevers in the South. The fevers on the Rio Grande at this time are so constantly attended with disordered stomach and bowels, that the morphia is indicated in almost every case requiring quinine.

As I before observed, our regiment took up the line of march on the 27th of July. We were under the necessity of leaving twenty-five sick men at the hospital in Memphis; most of them, however, were convalescent and followed in a few days. The direction of our march was a little south of west, passing entirely through Arkansas from north-east to south-west, and through the whole of Texas in the same direction. A geological and topographical history of this country would be a valuable and interesting work. My duties in another capacity were too arduous to allow much time for such investigations. From Memphis to Little Rock, the capital of Arkansas, the road passes through a low, flat, marshy country, pregnant with local causes of disease; and our troops suffered more, perhaps, in performing that distance of one hundred and fifty miles than on any other portion of the route. The weather was excessively warm, the roads dusty, and the water disgustingly bad; this was felt most severely in the Mississippi swamp, a distance of forty-five miles. Here, exposure to the sun, bad water and imprudence in diet brought on many cases of severe diarrhœa; the form of fever was principally malignant intermittent. I found it necessary not only to give as much as thirty grains of quinine at a dose, but to assist it with stimulants, external irritants, etc. Congestion was the symptom most to be feared, and there was no time to "prepare for quinine;" it must be given at once, and boldly given, or the patient was lost. It is astonishing how soon the worst cases recovered. Our conveniences for transporting the sick consisted of an ambulance fixed upon springs, and as many common wagons as were required. A great many preferred remaining on horseback, on account of the closeness of the wagons and the extreme heat. Measles on this account gave us much trouble; but notwithstanding all these inconveniences, we arrived at Little Rock in good time and without the loss of a man.

On this route an accidental case of surgery occurred, which, from its novelty in one particular, may be worth mentioning: A soldier, in taking a carbine from among some bridles and saddles, accidentally discharged it, the ball taking effect upon his comrade, who was standing so near that his clothes were set on fire. It entered the anterior inferior part of the axilla, ranged upward, and passed out at the anterior edge of the scapula. Not being near at the time myself, the assistant surgeon, Dr. Washington, examined and dressed the wound. He reported to me that he thought the ball had passed above the axillary artery, fracturing the humerus at the head or neck. I had only time the next day to make a superficial examintion of the wound, which I did without taking the bandages off. I discovered that the bone was evidently fractured, but at what point I could not determine, in the situation in which the arm then was. Dr. Washington was left in charge of the case, and reported to me, a short time afterwards, that on a more minute examination, he had discovered that the humerus was fractured, not at the head or neck as he had at first supposed, but three or four inches below, near the insertion of the latissimus dorsi and pectoralis major muscles; the axillary artery had remained uninjured, and could be distinctly felt pulsating when the finger was introduced into the wound. Suppuration was free, and the wound healed about the time that the bone united. In this case, the ball passed below the artery, but what broke the bone so far from the place at which it entered I do not clearly perceive; there was no fall or jar of any kind except that made by the ball itself.

At Little Rock we remained five or six days, recruiting; here the diseases assumed a milder form, intermittents being most common, and on the whole the number of cases was considerably diminished. Our march was now through a high, broken country, to Washington, in Arkansas, a distance of one hundred and twenty miles. The health of the regiment continued to improve until we crossed Red River, at Fulton, and encamped upon the edge of the swamp. Here my notes show a large increase in the number of cases of malignant intermittents and remittents, requiring even a more vigorous treatment than before. Congestive fever, in its worst form, prevailed at this time; remittents were also more obstinate, and required a more liberal use of mercurials. As the sick had to be hauled, these were given invariably at night, and in every case of fever where there was a remission, quinine was given in the morning, and repeated once or twice if necessary, regardless of the effect of the purgative given the night before. The condition of the bowels was attended to after the fever was broken, and quinine would do this whether the bowels were acted upon or not.

It may be worthy of remark, and I noticed it in a hundred instances, that though intermittent or remittent fever often preceded measles, it never accompanied or succeeded it during the march; diarrhœa, on the contrary, almost invariably followed it. The number of cases of fever occurring during the march through Texas was immense; this may be attributed in a great measure to exposure to the rays of an ardent sun during the day, and encamping upon the marshes and swamps of creeks and rivers at night. In the prairies, the heat of the sun was most intense, and was especially oppressive to persons from the North.

About the first of September, many cases of jaundice occurred; this was often though not always preceded by an attack of fever. The complaint was never serious, but excessively annoying to the patient, in consequence of the languor and general feeling of indisposition which it induced. It was almost invariably accompanied by a ravenous appetite. An emetic was given in the onset of the disease; this was followed by a brisk purgative, and then vegetable tonics were relied on to effect the cure. This course I persevered in for some time, but the cases did not recover as rapidly as we like to see them in the army. Another remedy must be sought for, more certain and rapid in its effects. Iodine struck me as coming nearer to fulfilling all the indications than any other I could think of, particularly the proto-iodide of mercury. This was given in the form of pill, in combination with rhubarb and aloes, twice a day, each dose containing two grains of the proto-iodide. My expectations were more than realized; indeed, it acted like a charm, and I had no further trouble with the disease.

I have now in a hurried and rather superficial manner referred to all the diseases we had to contend with in large numbers; of course there were many other isolated cases of rheumatism, dropsy, neuralgia, paralysis, etc., which recovered under the usual treatment. In giving the treatment of the different diseases encountered, I have left out all such remedies as are used merely as auxiliaries, and confined myself to such as were mainly relied on. Indeed, on a march through a wild country like that through which we passed, where both sick and well had to move at the sound of the horn in the morning, there was no time nor opportunity allowed for dealing in those minor remedies, so often used in domestic practice, more with a view of amusing the patient than with a hope of benefiting him.

Three deaths occurred on the road, one from each section of the State. No perceptible difference could be observed in the number or violence of the cases in the different companies after getting fairly into a southern climate, for there cannot now be found three men from any company who have not been under the care of the surgeon.

At Lavacca, a small town on the Matagorda Bay, now used as a depot for one division of the army, the regiment remained stationary eight or ten days; here my own health became so feeble that I obtained permission to go on to Matamoras by water. On the first of November the troops crossed the Rio Grande, and encamped on the bank of the river five miles above town.

Here the diseases have been chiefly of an intermittent form, accompanied in almost every case by gastric and intestinal derangement, and, owing to this, more difficult to treat than heretofore. Quinine alone almost invariably vomits; but rarely ever does so in combination with morphia, particularly if the precaution is used of applying a mustard plaster over the epigastric region at the time it is given. Owing to the frequent changes in the weather, relapses often occur, and the stage of convalescence is always protracted. Mercurials are often indicated, and are used with great advantage.

December, 1846.

